# Microbiota Profiles of Hen Eggs from the Different Seasons and Different Sectors of Shanghai, China

**DOI:** 10.3390/microorganisms11102519

**Published:** 2023-10-09

**Authors:** Haiyan Gong, Yingqing Ma, Min Wang, Yumeng Gu, Ruipeng Deng, Bo Deng, Dongsheng Feng, Yiyi Han, Rongsheng Mi, Yan Huang, Yan Zhang, Weiyi Zhang, Zhaoguo Chen

**Affiliations:** 1 Key Laboratory of Animal Parasitology of Ministry of Agriculture, Shanghai Veterinary Research Institute, Chinese Academy of Agricultural Sciences, Shanghai 200241, China; gonghaiyan@shvri.ac.cn (H.G.);; 2 Food Quality Supervision, Inspection and Testing Center of the Ministry of Agriculture and Rural Affairs (Shanghai), Shanghai Center of Agricultural Products Quality Safety, Shanghai 201708, China

**Keywords:** eggshell, content, microbiota, *Staphylococcus*

## Abstract

Hen eggs are one of the most popular foods worldwide, and their safety is critical. Employing 16S rRNA full-length sequencing is an effective way to identify microorganisms on or in eggs. Here, hen eggs collected from poultry farms over four seasons, as well as from markets in Shanghai, were analyzed with third-generation sequencing. *Firmicutes* (44.46%) and *Proteobacteria* (35.78%) were the two dominant phyla, and *Staphylococcus*, *Acinetobacter*, *Aerococcus*, *Psychrobacter*, and *Lactobacillus* were the dominant genera. The dominant genera on the eggshell surfaces from the farms varied with the seasons, and the highest contamination of *Staphylococcus* (32.93%) was seen in the eggs collected during the summer. For the market samples, *Pseudomonas* was the most abundant in content, with *Staphylococcus* being the most-often genera found on the eggshell surfaces. Moreover, several potential pathogenic bacteria including *Riemerella anatipestifer* (species), *Klebsiella* (genus)*,* and *Escherichia/shigella* (genus) were detected in the samples. The results revealed the impacts of weather on the microbiota deposited on an eggshell’s surface, as well as the impacts due to the differences between the contents and the surface. The results can help disinfect eggs and guide antibiotic selection.

## 1. Introduction

Eggs are a cheap and nutritious food, with more than 1.2 billion eggs consumed worldwide annually. China is the country with the highest levels of consumption, and it has been found to account for 35% of global egg production [1], which highlights the importance of food safety in hen eggs. Moreover, the international export of eggs from China is low because of concerns about food safety and quality [2]. In order to enhance the food safety of eggs and address this issue, it is essential to conduct an initial investigation into the current condition of eggs.

Egg safety concerns are associated with drug residues, illegal or excessive additives, and microbial contamination. The first two are fixed and can be easily avoided by strict laws and regulations as well as farming practices, but the later may dynamically change due to vertical and horizontal transmission during farming and transport processes [3]. Eggs and egg products are the known food source of 44% of the human cases of salmonellosis in the European Union [4]. To maintain egg safety from a microbial aspect, the United States Food and Drug Administration (FDA), the United States Department of Agriculture (USDA), and the European Union have established a series of rules (e.g., the Egg Safety Rule, Egg Safety Regulations, Egg Grading, and the European Union’s Regulation No 2160/2003). These rules include criteria for monitoring and testing for *Salmonella Enteritidis* in hens, eggs, and their environments, which ensures egg quality standards and safety. National health standards and rules [2] (GB 2749-2003, SN/T 0422-2010, and NY/T 754-2011) have also been established in China for several microorganisms, including *Salmonella*, *Shigella*, *Staphylococcus aureus*, etc. The bacterial contamination of eggshells at the time of collection affects the final products and may cause foodborne illnesses; thus, the high bacterial load of floor eggs means they cannot be eaten [5]. In addition, the pathogenic bacteria and the microorganisms that are deposited on eggshells, in egg whites, and in egg yolks can affect egg quality and shelf life. 

Recently, the V3-V4 variable region or full-length sequencing of 16S rRNA genes have been used to explore the microbiota in the guts of chickens [6], in their feces [7], on eggshells [8], and in egg yolks [9]. The results showed that the bacteria on eggshells changed as a function of the area [8] and the breed of the chicken [10], as well as due to farming practices, including the cages [10], food, and water used [3]. The microbiota in egg contents were tightly associated with the storage time [11]. However, information about the impacts of the different seasons on the microbiota on eggshells remains scarce, and these impacts involve changes in temperature, humidity, rainfall, light, etc. Moreover, it is unclear whether the bacterial loads on eggshells from markets are similar to those in the interiors of hen eggs.

Here, hen eggs from local farms, as well as those from markets, were studied with full-length 16S rRNA sequencing to provide basic data on market quality and improve food safety in Shanghai, China. 

## 2. Materials and Methods

### 2.1. Egg Samples Collection

Fresh hen eggs from poultry farms in suburban Shanghai were collected in the autumn of 2020 through to the summer of 2021 (the sampling scheme is shown in Supplementary Table S1). Simultaneously, hen eggs were randomly collected from open-air agricultural product markets. All hen eggs were sealed in sterile plastic bags with sterilized gloves [8] and brought to the lab within 24 h for sample preparation. Batches of five eggs were collected on the same days from the same places. Eggshell washes as well as the contents were pooled. Each eggshell was washed with a pre-sterilized cotton swab and immersed in a tube with 5 mL of 0.85% NaCl according to a previous study’s description [7]. One swab was applied for every five eggshells, and the liquid was then collected into a tube as one sample for DNA extraction. After flame sterilization of the eggs end surfaces, the eggshells were broken and the five egg contents were pooled, mixed completely, and 40 mL was sampled for further analysis. 

### 2.2. DNA Extraction

After condensing the microorganisms by thorough mixing and a subsequent centrifugation (12,000 rpm for 10 min), the total bacterial DNA from the eggshell or content samples was extracted using a TGuide S96 Magnetic Soil/Stool DNA Kit (Tiangen Biotech, Beijing, China) according to the manufacturer’s manual, and the quality was evaluated on a 1.8% gel. DNA from each sample was diluted to 1 ng/µL using sterile water and subjected to 16S rRNA full-length sequencing at TIANGEN Biotech (Beijing) Co., Ltd. 

### 2.3. The 16S rRNA Full-Length Sequencing

The specific forward primer 27F (5′-AGRGTTTGATYNTGGCTCAG-3′) and reverse primer 1492R (5′-TASGGHTACCTTGTTASGACTT-3′) that targeted the full 16S rRNA gene of the bacteria were synthesized with the barcodes attached, followed by PCR amplification and then product purification, quantification, and normalization to form the single-molecule real-time (SMRT) Bell sequencing libraries. The libraries were then treated with high-throughput sequencing (HTS) using a PacBio Sequel system (PacBio) [12], which resulted in data in bam format. These data were exported as CCS files via the SMRTlink analysis software (SMRTLink V12.0, Pacific Biosciences of California, Inc., San Diego, CA, USA). According to the barcode sequences, different samples were identified.

### 2.4. The 16S rRNA Sequence Data Preprocessing, Denoising, and Clustering

The data quality was evaluated using parameters including the number of SeqNum sequences, the amount of SumBase data, the N50 length, the N90 length, and the average length, followed by cutting the primer fragments in the sequences using cutadapt. The sequencing data were then filtered, de-noised, and de-chimerized by DADA2 to obtain the de-duplication amplicon sequence variants (ASVs) or operational taxonomic units (OTUs). The ASV/OTU data were standardized using QIIME2 software (V.2021.4, https://github.com/qiime2/qiime2) [13] to perform the subsequent species and diversity index analysis.

### 2.5. The Species Annotation

The bacterial 16S rRNA gene sequences were blasted against the SILVA reference database (Release 138, http://www.arb-silva.de (accessed on 27 August 2020). For the characteristic sequence of each ASV or the representative sequence of each OTU, the default parameters were used in the QIIME2 software, and a pre-trained Naive Bayes classifier was used to annotate the species [14]. The community composition of each sample was determined at each level (phylum, class, order, family, genus, and species), and the abundances at different classification levels were evaluated. 

### 2.6. α-Diversity Analysis

The alpha diversity was analyzed through six indices, including the Observed-species, Chao1, Shannon, Simpson, ACE, and Good-coverage indices, and these were calculated with QIIME2 and presented with R software (V4.0.3, https://cran.r-project.org/src/base/R-4/).

### 2.7. β-Diversity Analysis

The beta diversity analysis was performed using QIIME2 software to compare the similarity between the different samples with respect to species diversity, and the analysis included a PCoA analysis, an NMDS analysis, Adonis, and a UPGMA analysis using four algorithms, including binary Jaccard, Bray-Curtis, weighted unifrac, and unweighted unifrac, to calculate the distances between the samples.

### 2.8. Statistical Analysis

The Venn diagrams at the ASV level and a bar plot at the relative abundance level were constructed using ImageGP (https://www.bic.ac.cn/ImageGP/index.php/Home/Index/VennDiagramhtml (accessed on March 2017). The top 50 with the average abundance at each taxonomic level were extracted and clustered according to the composition and relative abundance of each sample or group to form the heatmap. Based the abundance data, the dynamic co-occurrence network was constructed [15] using the SparCC algorithm (V2, https://github.com/TankMermaid/sparcc-2). Metastats and STAMP software (Tiangen Biotech, Beijing, China) were utilized to confirm differences in the abundances of the individual taxonomies or function annotations between the two groups. ANOVA tests (included in R software) were performed based on the Bray–Curtis dissimilarity distance matrices to identify the differences in the microbial communities between the two groups.

## 3. Results

### 3.1. Overview of All the Bacterial Sequences

We obtained 97 libraries composed of 47 samples from native farms and 50 from markets (25 from eggshells and 25 from the contents). The fresh eggs from the poultry farms had bacterial DNA in the egg contents in quantities that were too small to be detected.

The raw reads in the 97 samples ranged from 4771–139,555, with an average of 10,027 reads. The ASVs ranged from 17–3108. A search of the SILVA 138 database showed that 14,842 ASVs could be annotated with a proportion of 99.8% at the phylum level, 96.27% at the genus level, and 59.14% at the species level. A total of 47 phyla including 978 genera and 1102 bacteria species were detected in the 97 egg samples. *Firmicutes* (44.46%), *Proteobacteria* (35.78%), and *Bacteroidota* (8.13%) were the dominant phyla (with a relative abundance of over 0.5% in each sample, Table 1). *Bacilli*, *Gammaproteobacteria,* and *Bacteroidia* were dominant at the class level. *Lactobacillales*, *Staphylococcales*, and *Pseudomonadales* were dominant at the order level. *Staphylococcaceae*, *Moraxellaceae*, and *Aerococcaceae* were the dominant at the family level. *Staphylococcus*, *Acinetobacter*, and *Aerococcus* were the dominant at the genus level (Table 2). The dominant three species were uncultured-*Ornithobacterium*, *Staphylococcus sciuri,* and *Psychrobacter faecalis*. 

### 3.2. Comparison of the Microbiota on the Eggshells from the Different Seasons

**Figure 1:**
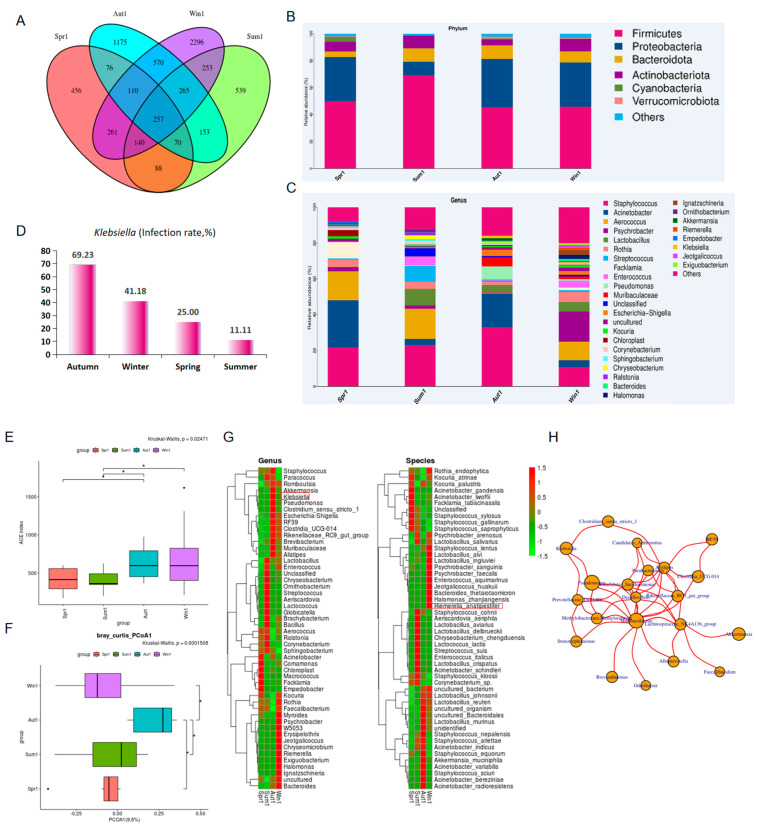
Microbiota analysis of the eggshell surface samples collected from the farms in Shanghai over the course of one year from the spring to the winter. Red, Spr1; green, Sum1; light blue, Aut1; purple, Win1. (**A**) The Venn diagram. (**B**) Stacked bar charts comparing the relative abundances of the bacterial phyla of the eggshell bacterial communities for each season. (**C**) Stacked bar charts comparing the relative abundances of the bacterial genera of the eggshell bacterial communities for each season. (**D**) Contamination rate for *Klebsiella* on the eggshells across each season. (**E**) The α—diversity analysis of the microbiota on the eggshells for each season. (**F**) The β—diversity analysis of the microbiota on the eggshells for each season. (**G**) Heatmap clustered by the genera abundance (left panel) or the species abundance (right panel) of each group. *Riemerella anatipestifer* was indicated with a red frame. (**H**) Dynamic network formed among the microorganisms deposited on the eggshells. Spr1, spring; Sum1, summer; Aut1, autumn; and Win1, winter.

Due to the failure to obtain the bacterial DNA from the egg contents, only the bacteria on the eggshells were analyzed. There were 47 eggshell samples, including 8, 9, 13, and 17 samples collected during the spring, summer, autumn, and winter, respectively. In total, 6709 ASVs were clustered, and the four seasons shared 257 ASVs, as shown in the Venn diagram. Intriguingly, the unique ASVs increased from the spring to the winter (Figure 1A). Based on the relative abundance at the phylum level, *Firmicutes* presented as the most dominant phylum (52.54 ± 11.14% of the total phyla) followed by *Proteobacteria* (27.95 ± 11.90%), *Bacteroidota* (8.09 ± 2.81%), and *Actinobacteriota* (7.61 ± 2.35%). These four occupied 94.1–98.7% of the total phyla (Figure 1B). 

The top five genera detected on the fresh eggshell surfaces included *Staphylococcus*, *Acinetobacter*, *Aerococcus*, *Psychrobacter*, and *Lactobacillus.* These were similar to those in the total samples (Table 2 and Table 3). When the relative abundance of genera for each season were compared, *Staphylococcus* showed the highest relative abundance in autumn (32.92%), followed by summer (22.92%). *Acinetobacter* (26.43%) and *Psychrobacter* (16.91%) were highest in the spring and winter, respectively (Table 4 and Figure 1C). There was a decrease in the contamination rate (contaminated samples/all samples × 100%) of the uncultured *Klebsiella* on the eggshell surfaces from autumn (69.23%) to summer (11.11%, Figure 1D). 

The α-diversity analysis indicated that the autumn group was significantly higher than the spring and summer groups (*p* < 0.05), which suggested that the number of bacterial species present in autumn was more than in the spring and summer (Figure 1E). The β-diversity represented the similarities or differences in the microbiota community compositions of the samples. The PCoA analysis based on the Bray–Curtis distance indicated that the similarity in the autumn samples was significantly less than that in the other three seasons (Figure 1F). The bacteria, including *Alistipes,* the unclassified RF39 (phyla *Tenericutes*), *Clostridia UCG.014, Rikenellaceae RC9 gut group, Clostridium sensu stricto 1*, *Klebsiella,* and *Pseudomonas,* showed a high abundance in autumn (Figure 1G). These observations were supported by the predicted network analysis using the SparCC algorithm, which hinted at a positive co-occurrence among the genera (Figure 1H). We also detected a high abundance of *Riemerella anatipestifer* (28.7%) on the winter samples from a farm in the Chongming district (Figure 1G, right panel). 

### 3.3. Comparison of the Microbiota on the Hen Eggshell Surfaces to the Contents Collected from the Markets

**Figure 2:**
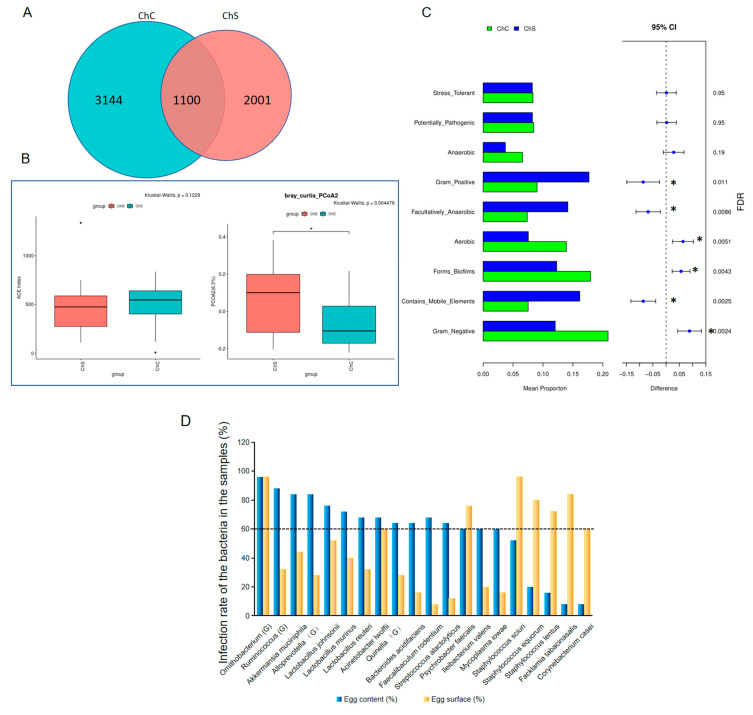

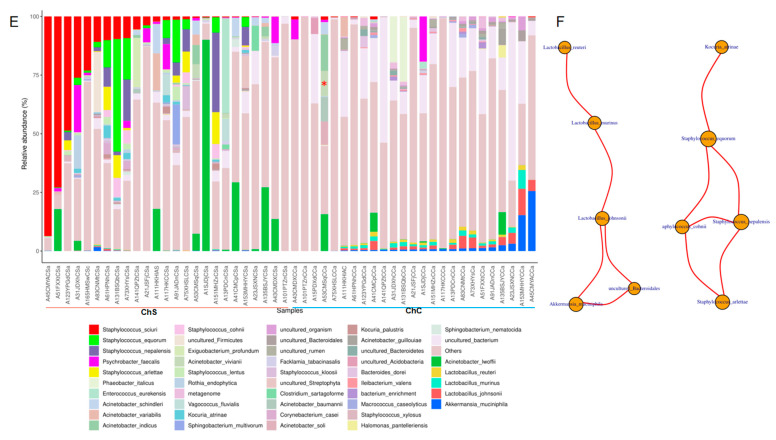
The microbiota analysis of the eggshell surfaces and contents collected from the markets in Shanghai. (**A**) The Venn diagram. (**B**) The ɑ-and β-diversity of the microbiome in the contents and on the eggshell surface samples. The stars showed the significant differences between two groups. (**C**) Top 10 different functions classified from the microorganisms in the contents and on the surfaces of the eggs. The significant different function features are shown with stars. (**D**) The bacterial species or genera with contamination rates of over 60%. The dashed line indicated the contamination rate of 60%. (**E**) Top bacterial species in the contents and on the eggshell surfaces, with the last five species as the top five in the egg contents. (**F**) Dynamic network formed among the microorganisms deposited in the egg contents (left panel) and on the eggshell surfaces (right panel). ChC, egg content; ChS, eggshell surface.

We constructed 50 egg sample pools, including 25 washing liquid samples from the eggshells and 25 corresponding egg content samples. In total, 6245 ASVs were clustered and annotated to 791 genera. The egg contents contained more ASVs (4244) than the surfaces (3101 ASVs) (Figure 2A). The α-diversity of the contents showed no significant difference from the surface samples, while the β-diversity was significantly lower than that of the surface samples (Figure 2B).

Interestingly, the STAMP analysis showed that the abundance of Gram-positive bacteria, facultative anaerobic bacteria, and bacteria containing mobile elements on the surfaces of the eggshells was significantly higher than that in the contents. Meanwhile, the abundance of Gram-negative bacteria (GNB), aerobic bacteria, and biofilm-forming microorganisms in the contents was significantly higher than that on the surface (Figure 2C).

At the genus level, *Staphylococcus* was dominant on the surface (30.84% vs 5.5% in the contents), followed by *Acinetobacter* (15.29%) and *Enterococcus* (7.43%, Table 5). *Pseudomonas* was the most abundant in the contents (8.47% vs. 0.32% on the surface) followed by *Lactobacillus* (6.89%) and uncultured bacteria (6.09%, Table 5). Twenty species or genera showed contamination rates of over 60% in the samples, with sixteen in the contents and eight in the surface samples (Figure 2D). Both the contents and the surface samples shared high occurrences of *Ornithobacterium* (Genus), *Psychrobacter faecalis*, and *Acinetobacter lwoffii.* Moreover, *Akkermansia muciniphila* and *Lactobacillus spp.*, including *L*. *murinus*, *L. johnsonii*, and *L. reuteri*, not only demonstrated high occurrences in the content samples (Figure 2D) but also ranked as the top five in relative abundance (Figure 2E). These bacterial species were predicted to be closely related (Figure 2F left panel). *Staphylococcus spp.*, including *S. equorum*, *S. nepalensis,* and *S. arlettae*, showed high relative abundances on the surface samples (Figure 2E), and they were positively connected (Figure 2F right panel).

### 3.4. Comparison of the Microbiota of the Eggshell Surfaces from the Farms and the Markets in Shanghai

**Figure 3:**
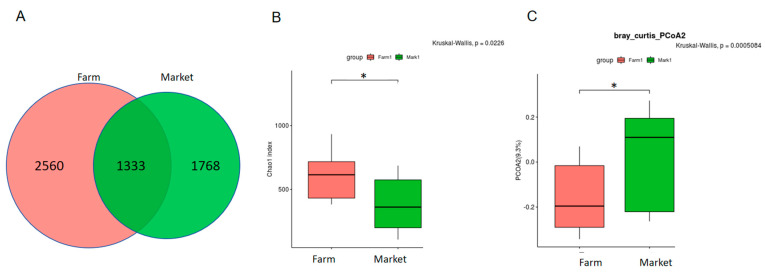
Microbiota analysis of the eggshell surface samples collected from the farms and markets. (**A**) Venn diagram. (**B**) α—diversity analysis of the microbiota on the eggshell surfaces. (**C**) β—diversity analysis of the microbiota on the eggshell surfaces. The stars showed the significant differences between two groups.

Eggs are exposed to air, hands, and containers during transport, and thus, the microbiota on eggshells can change dynamically. In the present study, market samples and farm samples were collected in summer and autumn for a comparison. Both groups shared the same top seven dominant phyla, with *Firmicutes* making up 50.87% and 51.36% of the total phyla in the farm and market samples, respectively (Table 6). However, the top 15 genera in the two groups were very different from each other except for the top two dominant genera *Staphylococcus* and *Acinetobacter* (Table 7). Here, 3893 ASVs were detected in the farm samples while 3101 ASVs were found in the market samples, which shared 1333 ASVs (Figure 3A). The microbiome from the farm samples demonstrated significantly higher α-diversity than that from the markets (Figure 3B), while the β-diversity showed the opposite trend (Figure 3C). 

### 3.5. Distribution of Escherichia/shigella in the Hen Egg Samples

**Figure 4:**
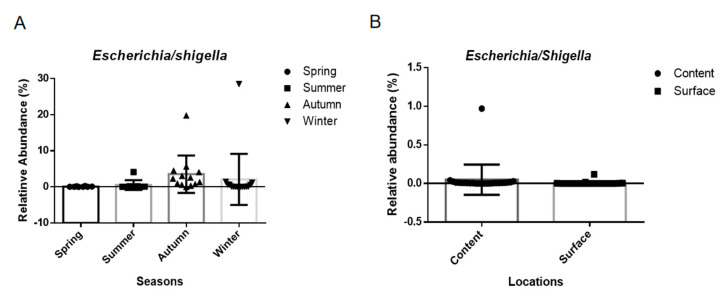
The relative abundance of *Escherichia/shigella* distributed in the different seasons (**A**) and in the different locations (**B**).

*Escherichia/shigella* is typically transmitted by a direct fecal–oral route, and it should not be detected in eggs according to the GB 2749 and NY/T 754 in China [2]. Therefore, surveillance for *Escherichia/shigella* was still necessary. We found that *Escherichia/shigella* could be detected on the surfaces and in the contents of the eggs but in low relative abundances (Figure 4). The distribution of the bacterium demonstrated no difference, but several fresh egg surface samples from autumn and winter contained *Escherichia/shigella* with a relative abundance of over 20% (Figure 4A). One pool in the egg contents from the markets contained a relative abundance of approximately 1% (Figure 4B).

## 4. Discussion

The bacteria on the eggshell surfaces demonstrated a high probability of trans-shell infection [16], and their growth was tightly associated with environmental conditions, including temperature and humidity [17]. The deposited bacteria on the fresh egg surfaces not only reflected the intestinal health statuses of the hens but also the pollution in the poultry farm environments. Therefore, the bacterial communities on the fresh chicken eggshells were investigated across the four seasons. The results showed that the autumn samples were more seriously contaminated than the spring and summer samples. In addition, during the winter season, the eggshell surfaces harbored greater varieties of microorganisms compared to the summer season (Figure 1E), which appeared to contrast with the results of a previous study showing that bacteria had higher counts on eggshells exposed to high temperatures compared to those exposed to low temperature [18]. This may be explained by the differences in the methods for laying and storage. In the present study, we obtained no data from the fresh egg contents because the extracted DNA was insufficient. To further explore contamination in the fresh egg contents, we introduced each sample from the contents onto TSA and blood agar plates. Nevertheless, no bacterial colonies were observed. 

Here, *Firmicutes* made up 50.87% of the total phyla on the fresh eggs and 51.36% on the market eggshell surfaces, which was consistent with a previous investigation on commercial eggshell surfaces [4]. Based on the findings that the microbial community in the chicken cloaca as well as in the oviduct was also largely dominated by *Firmicutes* [19,20], the vertical microbiota transmission was again shown to be critical for the contamination of eggshells and egg safety as described by Trudeau et al. [7]. However, *Proteobacteria* ranked second in the total samples, which was different from a similar study on eggshells from the Shanxi province that showed a majority made up of *Firmicutes* and *Actinobacteriota* [8]. These differences may have been due to the different weather or different kinds of hens. The main bacteria producing malodorous trimethylamine in ceca were included in *Firmicutes* and *Proteobacteria* [21], and thus, this suggested that the chicken eggs produced in Shanghai were more likely to spoil and should therefore be treated before sale or kept for a shorter time before cooking. 

Intriguingly, when the microbiomes were compared to the egg surfaces, Gram-negative, aerobic, and biofilm-forming microorganisms in the contents were significantly abundant, while Gram-positive, facultative anaerobic bacteria and bacteria containing mobile elements were less abundant. We wondered why Gram-negative and biofilm-forming species were more abundant in the egg contents. Bacterial biofilms are communities of cells that are attached to surfaces and to each other, which can protect the imbedded bacteria from the host’s defense system to improve their survival potential [22]. The biofilms formed by *S. mutans* were shown to be highly resistant to harsh environments, host immunity, antimicrobial treatments, and lysozyme [23]. Therefore, we concluded that the biofilm-forming bacteria including *Pseudomonas* may have had more opportunity to overcome the lysozymes and invade the egg whites. As shown in this study, *Pseudomonas* had the highest abundances in the market egg contents. 

*Enterobacteriaceae* account for approximately 80% of GNB isolates. The genera/species that frequently affect humans are *Escherichia*, *Proteus*, *Enterobacter*, *Klebsiella*, *Citrobacter*, *Yersinia*, and *Salmonella.* GNB have two membranes: external and internal [24]. The former protects GNB from many antibiotics (including penicillin), detergents (peptidoglycan, which usually damages the inner membranes of cells), and lysozymes. Aminoglycosides, monolactams (aztreonam), and ciprofloxacin are drugs that specifically target Gram-negative organisms. Moreover, GNB can reach almost all systems in the human body and are apt to cause disease in humans [24] We found a higher abundance of GNB in the egg contents, which requires further research to improve egg safety. 

*Klebsiella* spp. is an opportunistic *Enterobacteriaceae* that is highly pathogenic to poultry, livestock, and humans, and it is commonly found throughout China [25,26] as well as in other countries [27,28]. Here, *Klebsiella* contamination on the eggshell surfaces from the poultry farms was detected, with a season-dependent occurrence rate. The highest infection rate was in autumn and the lowest rate was in summer. This could explain the high rates of respiratory diseases in autumn and winter in chickens that have been vaccinated against other respiratory disease-causing pathogens, including the Newcastle disease vaccine and infectious bursal disease vaccines. *Klebsiella* spp. can survive for a long time in a henhouse, and the bacteria carried in poultry, its products, such as edible eggs and broiler chickens, and its environment may pose a serious risk to consumers. Moreover, the antibiotic-resistant isolates of *Klebsiella* spp. may transfer resistance genes to other microorganisms—especially genes conferring resistance to carbapenems [28]. Therefore, for the health of both chickens and consumers, surveillance and subsequent controls for this bacterium are supposed to be significant and necessary. In addition, this seasonal distribution trend of *Klebsiella* was quite consistent with an investigation on *K. pneumoniae* infection carried out in a hospital near Shanghai [29]. This suggested that poultry, its products, and its environment may be the source of human infection with *K. pneumoniae*, which deserves further investigation.

Moreover, we detected a potential pathogen, *R. anatipestifer,* on the hen eggshells from a winter sample by 16S rRNA full-length sequencing. *R. anatipestifer* is a widely distributed and highly infectious bacterial pathogen for ducklings, goslings, turkeys, and other poultry. It causes pericarditis, perihepatitis, airsaculitis, etc. in its host, which has led to severe economic damage to the duck breeding industry. Moreover, since 2019, chicken-derived *R. anatipestifer* has been isolated from sick chicken organs (tarsal joints, sinuses, liver, cheese-containing fallopian tubes, brains, and dead embryo egg fluid) [30]. In chickens, infection with *R. anatipestifer* may not only cause high mortality but also facilitate secondary infections by other bacteria or viruses. In this study, we detected *R. anatipestifer* on the normal eggshells, which may have indicated contamination of the environment by *R. anatipestifer* due to the mixed rearing of ducks and chickens. Although chicken-derived *R.anatipestifer* was not isolated in the samples and no clinical case has been reported in Shanghai, it is imperative to proactively trace a source for early screening and prevention. This is particularly crucial due to the novelty of this pathogen in chicken-derived infections.

*Escherichia/shigella* has been widely detected and described in gut microbiota and is a negative indicator for human diseases [31,32]. Recently, it has been reported as pathogenic or spoilage bacteria that appears on eggshell surfaces as well as in feces [7]. We also found *Escherichia/shigella* in the egg contents in low abundances, except for a few outliers. However, this should be studied further for egg food safety.

## 5. Conclusions

In summary, the microorganisms on eggshells and in eggs were investigated by 16S rRNA full-length sequencing. Our results indicated that the microbiomes varied during the four seasons. Moreover, the content of the eggs from the markets were contaminated with *Pseudomonas*, *Lactobacillus*, and others. The results underscored the importance of strengthening inspection. Consumers should completely cook eggs to reduce risks.

## Figures and Tables

**Table 1:** The top 10 dominant phyla of the microbiota detected in all samples.

Top	Phylum	Relative Abundance (%)
1	*Firmicutes*	44.46
2	*Proteobacteria*	35.78
3	*Bacteroidota*	8.13
4	*Actinobacteriota*	5.88
5	*Verrucomicrobiota*	1.31
6	*Cyanobacteria*	0.84
7	*Desulfobacterota*	0.72
8	*Planctomycetota*	0.60
9	*Acidobacteriota*	0.49
10	*Patescibacteria*	0.38

**Table 2:** The top 15 dominant genera of the microbiota detected in all samples.

Top	Genus	Relative Abundance (%)
1	*Staphylococcus*	19.05
2	*Acinetobacter*	11.02
3	*Aerococcus*	5.65
4	*Psychrobacter*	5.24
5	*Lactobacillus*	4.30
6	*Pseudomonas*	3.42
7	*Enterococcus*	2.79
8	*uncultured*	2.65
9	*Muribaculaceae*	2.53
10	*Enterobacter*	2.43
11	*Escherichia-Shigella*	2.30
12	*Rothia*	2.19
13	*Unclassified*	2.00
14	*Streptococcus*	1.24
15	*Facklamia*	1.21

**Table 3:** The top 15 dominant genera of the microbiota detected in samples from the farms.

Top	Genus	Relative Abundance (%)
**1**	*Staphylococcus*	20.59
**2**	*Acinetobacter*	11.51
**3**	*Aerococcus*	10.01
**4**	*Psychrobacter*	7.28
**5**	*Lactobacillus*	4.86
**6**	*Rothia*	4.09
**7**	*Enterococcus*	2.63
**8**	*Streptococcus*	2.33
**9**	*Pseudomonas*	2.18
**10**	*Facklamia*	2.14
**11**	*Muribaculaceae*	1.94
**12**	*Escherichia-Shigella*	1.80
**13**	*uncultured*	1.42
**14**	*Kocuria*	1.16
**15**	*Halomonas*	0.91

**Table 4:** The top 15 dominant genera of the microbiota detected in samples collected over the four seasons.

Top	Spring		Summer		Autumn		Winter	
	Genus	Relative Abundance (%)	Genus	Relative Abundance (%)	Genus	Relative Abundance (%)	Genus	Relative Abundance (%)
**1**	*Acinetobacter*	26.43	*Staphylococcus*	22.92	*Staphylococcus*	32.92	*Psychrobacter*	16.91
**2**	*Staphylococcus*	21.58	*Aerococcus*	16.86	*Acinetobacter*	18.95	*Staphylococcus*	10.76
**3**	*Aerococcus*	16.23	*Lactobacillus*	9.24	*Pseudomonas*	7.17	*Aerococcus*	10.22
**4**	*Facklamia*	9.13	*Streptococcus*	8.83	*Muribaculaceae*	5.07	*Rothia*	5.71
**5**	*Rothia*	4.17	*Enterococcus*	4.85	*Lactobacillus*	4.07	*Lactobacillus*	5.26
**6**	*Chloroplast*	3.39	*Unclassified*	4.61	*Escherichia-Shigella*	3.49	*Acinetobacter*	3.89
**7**	*Psychrobacter*	2.30	*Rothia*	4.01	*Rothia*	1.65	*Enterococcus*	3.88
**8**	*Kocuria*	1.42	*Acinetobacter*	3.53	*uncultured*	1.33	*Halomonas*	2.30
**9**	*Empedobacter*	1.38	*Chryseobacterium*	2.17	*Klebsiella*	1.31	*Ignatzschineria*	2.08
**10**	*uncultured*	1.30	*Corynebacterium*	1.91	*Akkermansia*	1.18	*Escherichia-Shigella*	2.08
**11**	*Ralstonia*	1.03	*Psychrobacter*	1.87	*Romboutsia*	0.99	*uncultured*	2.00
**12**	*Corynebacterium*	1.03	*Ralstonia*	1.56	*Sphingobacterium*	0.84	*Riemerella*	1.60
**13**	*Sphingobacterium*	0.94	*Ornithobacterium*	1.48	*Enterococcus*	0.80	*Muribaculaceae*	1.55
**14**	*Comamonas*	0.70	*Sphingobacterium*	1.14	*Bacteroides*	0.79	*Kocuria*	1.40
**15**	*Macrococcus*	0.55	*Kocuria*	0.97	*Kocuria*	0.76	*Facklamia*	1.39

**Table 5:** The top 15 dominant genera of the microbiota detected on the eggshell surfaces and in the egg contents.

Top	Surface		Content	
	Genus	Relative Abundance (%)	Genus	Relative Abundance (%)
**1**	*Staphylococcus*	30.84	*Pseudomonas*	8.47
**2**	*Acinetobacter*	15.29	*Lactobacillus*	6.89
**3**	*Enterococcus*	7.43	*uncultured*	6.09
**4**	*Psychrobacter*	5.33	*Acinetobacter*	5.57
**5**	*Enterobacter*	5.28	*Staphylococcus*	5.50
**6**	*Aerococcus*	3.01	*Escherichia-Shigella*	4.82
**7**	*Muribaculaceae*	2.40	*Enterobacter*	3.86
**8**	*Exiguobacterium*	1.94	*Muribaculaceae*	3.78
**9**	*Granulicatella*	1.94	*Sphingomonas*	3.53
**10**	*Sphingobacterium*	1.91	*Unclassified*	3.51
**11**	*Kocuria*	1.55	*Skermanella*	2.97
**12**	*uncultured*	1.52	*Stenotrophomonas*	2.69
**13**	*Klebsiella*	1.39	*Nautella*	1.88
**14**	*Unclassified*	1.22	*Cutibacterium*	1.56
**15**	*Akkermansia*	1.19	*Bacteroides*	1.39

**Table 6:** The top 10 dominant phyla of the microbiota detected on the eggshell surfaces from the farms and the markets.

Top	Farm		Market	
	Phylum	Relative Abundance (%)	Phylum	Relative Abundance (%)
1	*Firmicutes*	50.87	*Firmicutes*	51.36
2	*Proteobacteria*	29.29	*Proteobacteria*	32.70
3	*Bacteroidota*	8.34	*Bacteroidota*	5.33
4	*Actinobacteriota*	7.72	*Actinobacteriota*	4.26
5	*Verrucomicrobiota*	0.73	*Verrucomicrobiota*	1.15
6	*Cyanobacteria*	0.90	*Cyanobacteria*	0.82
7	*Desulfobacterota*	0.12	*Campilobacterota*	0.14
8	*Planctomycetota*	0.43	*Fusobacteriota*	0.09
9	*Acidobacteriota*	0.33	*Patescibacteria*	0.08
10	*Patescibacteria*	0.38	*Myxococcota*	0.07

**Table 7:** The top 15 dominant genera of the microbiota detected on the eggshell surfaces from the farms and the markets.

Top	Farm		Market	
	Genus	Relative Abundance (%)	Genus	Relative Abundance (%)
**1**	*Staphylococcus*	20.59	*Staphylococcus*	27.91
**2**	*Acinetobacter*	11.51	*Acinetobacter*	11.66
**3**	*Aerococcus*	10.01	*Enterococcus*	8.56
**4**	*Psychrobacter*	7.28	*Psychrobacter*	6.24
**5**	*Lactobacillus*	4.86	*Enterobacter*	3.79
**6**	*Rothia*	4.09	*Aerococcus*	2.95
**7**	*Enterococcus*	2.63	*Kocuria*	1.84
**8**	*Streptococcus*	2.33	*Muribaculaceae*	1.84
**9**	*Pseudomonas*	2.18	*Romboutsia*	1.82
**10**	*Facklamia*	2.14	*Exiguobacterium*	1.59
**11**	*Muribaculaceae*	1.94	*uncultured*	1.55
**12**	*Escherichia-Shigella*	1.80	*Granulicatella*	1.53
**13**	*uncultured*	1.42	*Sphingobacterium*	1.39
**14**	*Kocuria*	1.16	*Lactobacillus*	1.33
**15**	*Halomonas*	0.91	*Kosakonia*	1.11

## Data Availability

Raw sequences of the 16S rDNA full lengths in FASTQ file format have been deposited in the NCBI’s Sequence Read Archive (SRA) under the BioProject accession number PRJNA1010744, with the sample ID from SAMN37196364 to SAMN37196460.

## Supplementary Materials

The following supporting information can be downloaded at: https://www.mdpi.com/article/10.3390/microorganisms11102519/s1, Table S1: The egg sampling scheme in different districts and in different seasons.
