# Supplementary material for: Microbiota Profiles of Hen Eggs from the Different Seasons and Different Sectors of Shanghai, China
Source: Microorganisms. 2023 Oct 9;11(10):2519. doi: 10.3390/microorganisms11102519 (PMC10609546; doi:10.3390/microorganisms11102519)
Supplement: Supplementary file 1 [file microorganisms-11-02519-s001.zip › microorganisms-2617643-supplementary.pdf]

Supplementary Table S1 The egg sampling scheme in different districts and in different seasons

**Collection time**	**Districts**						
	Fengxian	Chongming	Jiading	Jinshan	Songjiang	Pudong	Total numbers
Septemper 2020	6	2	1	4	0	0	13
November and December 2020	9	2	1	3	1	1	17
March 2021	2	1	1	3	1	0	8
June 2021	5	0	1	2	1	0	9
